# Multimerized epitope tags for high-sensitivity protein detection

**DOI:** 10.1093/g3journal/jkaf070

**Published:** 2025-04-07

**Authors:** R Steven Stowers

**Affiliations:** Department of Microbiology and Cell Biology, Montana State University, Bozeman, MT 59717, USA

**Keywords:** *Drosophila*, vGlut, synaptic vesicle, epitope tag, FlyBase

## Abstract

A detailed understanding of the function of a gene requires knowledge of the cellular and subcellular distribution of its encoded protein(s). For proteins expressed at low levels, antibodies that recognize single epitopes may not be sufficient for visualizing expression. To enhance the sensitivity of protein detection, tandem repeat multimers of the commonly used epitope tags V5, HA, MYC, FLAG, ALFA, and OLLAS were developed that encode up to 80X copies of each tag, an 8-fold increase over currently available options for epitope multimer tagging. As proof-of-principle, conditional alleles of vGlut containing the 40XV5 and 40XMYC epitope tag multimers were validated *in vivo* in *Drosophila*. Both epitope-tagged proteins were determined to exhibit synaptic localization in the adult brain and larval neuromuscular junction similar to that of endogenous vGlut. They were also conditionally expressed in subsets of adult brain neurons and observed to exhibit robust, easily detectable expression in presynaptic terminals even in single neurons. These highly multimerized epitope tags will facilitate any type of experiment using antibody detection of proteins that would benefit from enhanced sensitivity.

## Introduction

Proteins mediate biological function. To understand biology, it is thus imperative to understand proteins, including their temporal and spatial expression patterns at both the cellular and subcellular levels. Established methods for detecting proteins include the use of antibodies that directly recognize proteins of interest (Fujita *et al.* 1982; Bekele-Arcuri *et al.* 1996; Uhlen *et al.* 2005; Hofbauer *et al.* 2009; Gong *et al.* 2016), or fusion of proteins of interest to fluorescent (Morin *et al.* 2001; Kelso *et al.* 2004; Weber and Koster 2013; Nagarkar-Jaiswal *et al.* 2015; Rodriguez *et al.* 2017; Doh *et al.* 2018; Hefel and Smolikove 2019; Avellaneda and Schnorrer 2022) or epitope tags (Deng *et al.* 2019; Tison *et al.* 2020) (Carrington *et al.* 2022; Igreja *et al.* 2022; Lim *et al.* 2022). While fluorescent protein fusions may be directly visualized via endogenous fluorescence, in all 3 cases, detection typically involves primary antibodies and fluorophore or enzyme-tagged secondary antibodies.

Generation of protein-specific antibodies is cumbersome, time-consuming, not always successful, and may even lead to unreproducible results (Rhodes and Trimmer 2006; Herkenham *et al.* 2011; Baker 2015; Bradbury and Pluckthun 2015). Even when successful, the sensitivity of detection can be limiting due to a single epitope in the case of monoclonal antibodies, or a few epitopes in the case of polyclonal antibodies. Hence, fusion of either a fluorescent protein (such as GFP) or epitope tag(s) is often the method of choice for visualizing protein localization. For assessing dynamic changes in protein localization, this is only possible with fluorescent proteins and depends on a protein of interest being expressed above a threshold level because endogenous fluorescence signal is often limiting. For many applications, however, knowledge of dynamic changes in protein distribution is not required, and epitope tagging is the preferred method of protein detection.

Epitope tags are advantageous for several reasons including: (1) they are small, typically 10–15 amino acids, and thus they have a low likelihood of altering the localization or function of a protein; (2) there are numerous distinct epitope tags for which antibodies are commercially available; (3) they have a high rate of success for a variety of applications (Western blot, immunohistochemistry, chromatin immunoprecipitation, expansion microscopy, etc.) and whether an antibody to a given epitope tag will work for a specific application is known beforehand (unlike newly generated protein-specific antibodies that may work for some applications but not others); (4) their specificity is known beforehand or can easily be assessed for background signal by testing on wildtype controls; (5) 10X epitope tag fusion proteins are available. The “spaghetti monster” (sm) 10X epitope tag proteins (Viswanathan *et al.* 2015), in particular, were a significant advance in the use of epitope tag multimers for enhancing protein detection sensitivity and their utility has facilitated numerous advances in understanding mechanisms of protein and cellular function (Munoz-Castaneda *et al.* 2021; Certel *et al.* 2022; Handler *et al.* 2023; Bonanno *et al.* 2024; Gao *et al.* 2024; Sanfilippo *et al.* 2024).

This report describes an expansion of the epitope tag multimerization repertoire from 10X, in the case of the spaghetti monster proteins, up to 80X for the 6 commonly used epitope tags V5, HA, MYC, FLAG, ALFA, and OLLAS, thus potentially enhancing protein detection sensitivity 8-fold. As proof-of-principle of their utility, the 40XV5 and 40XMYC epitope tag multimers were genome edited onto the Drosophila vesicular glutamate transporter vGlut and their expression was assessed in the adult brain, at the larval neuromuscular junction, and in individual neurons in the adult brain using conditional expression.

## Materials and methods

### Fly strains

Stocks from the Bloomington Drosophila Stock Certer (NIH P40OD018537) were used in this study. Previously generated fly strains include: *20XUAS-B2* and *n-syb-GAL4* (Williams *et al.* 2019); *nos-GAL4* RRID:BDSC_4442 (Tracey *et al.* 2000); *smFLAG-vGlut* RRID:BDSC_93891 (Certel *et al.* 2022); *vGlut^SS1^* RRID:BDSC_91246 (Sherer *et al.* 2020); *MB434B* (MBON-5/6) RRID:BDSC_68325 (Aso *et al.* 2014); *UAS-CD8-mCherry* RRID:BDSC_27392.

### Epitope tag multimer assembly

**Fig. 1.**
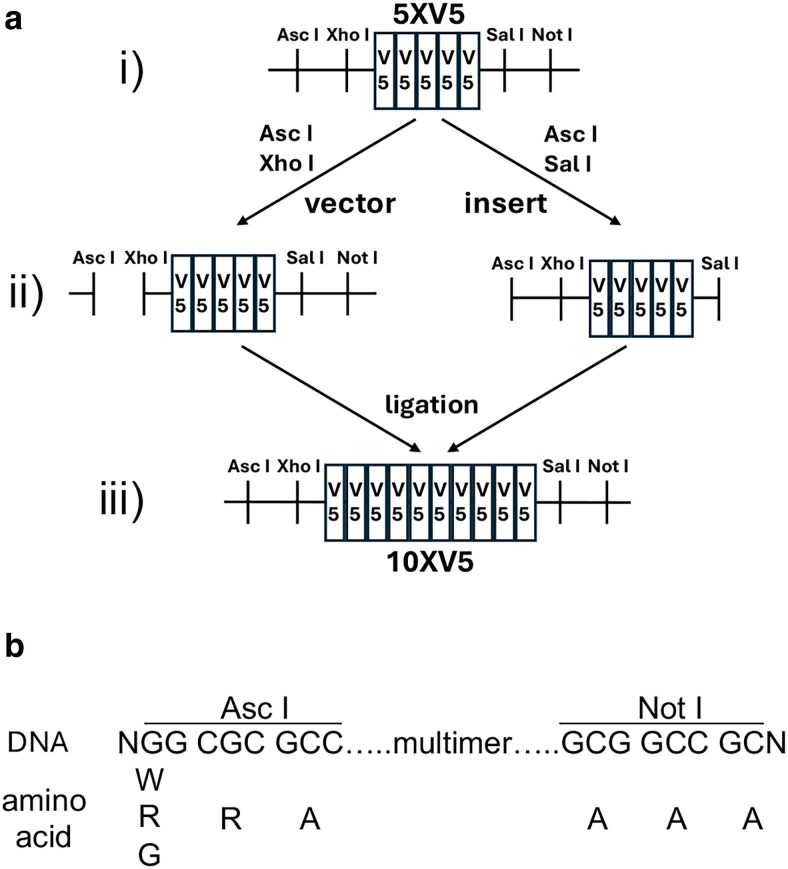
Epitope multimerization strategy. ai) Five tandem repeats of the V5 epitope tag were commercially synthesized in the *pUC57Kan* vector. The gene synthesis plasmids also included unique *Asc I* and *Xho I* restriction sites on the upstream side as well as unique *Sal I* and *Not I* restriction sites on the downstream side of the tandem repeats. aii) To double the number of V5 repeats, the plasmid was cut with *Asc I* and *Xho I* to generate the vector and separately with *Asc I* and *Sal I* to create the insert. The vector and insert were ligated together using T4 DNA ligase by virtue of the compatible cohesive ends of *Xho I* and *Sal I*. aiii) The resulting plasmid contains 10 in-frame tandem copies of the V5 epitope tag. The hybrid *Xho I*/*Sal I* site between the 5XV5 repeats is not recognized by either restriction enzyme, thus regenerating the original configuration of unique restriction sites in ai) and allowing the same cloning strategy to be iteratively repeated with a doubling of the epitope tag copy number with each round of cloning. This cloning strategy was sequentially repeated 4 times to produce plasmids with 10X, 20X, 40X, and 80X tandem epitope tag multimer repeats. This cloning strategy should also be generalizable for multimerizing other types of DNA sequences. b) Reading frames on either side of the epitope tag multimers. To be in frame with the epitope tag coding sequences, codons spanning the *Asc I* site include an immediately upstream nucleotide and the first 2 base pairs of the *Asc I* recognition sequence, NGG, followed by CGC and GCC. The reading frame on the *Not I* side includes the first three codons of the *Not I* recognition sequence, GCG, followed by GCC, and GCN where N is the next adjacent downstream nucleotide. Note that the open reading frame on the *Not I* side is open. Carboxy-terminal fusions should thus include a downstream STOP codon. The open reading frame on the downstream *Not I* side allows the incorporation of epitope tag multimers both at the amino-terminus as well as sites internal to the protein of interest.

5X epitope tag multimer plasmids were generated by gene synthesis in the *pUC57Kan* vector by Synbio. Sequential rounds of restriction cloning *Asc I*/*Sal I* inserts into *Asc I*/*Xho I* vectors as outlined in Fig. 1 were used to generate the 10X, 20X, 40X, and 80X epitope tag multimer plasmids. The epitope tag multimers were generally stable up to as least 20X in the *pUC57Kan* vector. However, it was empirically determined that some multimers were more stable in the vGlut donor plasmid and many of the epitope tag multimers were therefore assembled in this plasmid. All cloning steps utilized the *DH5α* bacterial strain.

### Guide RNA plasmid construction

*pCFD4* Guide RNA plasmids were generated as previously described (Port *et al.* 2014). The guide RNA plasmids and the guide RNAs they contain are as follows: *pCFD4-vGlut78*-TGTTTGTCTATTCGAGTT and CTAAGTGCATTGGAATAT; *pCFD4-vGlut910*-ATATATATCAGTGACCTA and GTTGAGTTCCATCGATGG.

### Donor plasmid construction

The starting point of the donor plasmids was gene synthesis of the homology arms that included mutated PAM sites, recombinase target sites, and the in-frame restriction sites *Asc I* and *Not I*. The STOP cassettes were added by Gibson cloning. The multimerized epitope tags were added by restriction enzyme cloning using the *Asc I* and *Not I* restriction sites. The complete sequence of the *B2RT-STOP-B2RT-vGlut-40XV5* donor plasmid is included in supplementary figures Supplementary Fig. 4a and 4b. The complete sequence of the *B2RT-STOP-B2RT-vGlut-40XMYC* donor plasmid is included in supplementary figures Supplementary Fig. 5a and 5b.

### Genome editing

For each genome edit, the donor plasmid was injected together with both *pCFD4* guide RNA plasmids into the *nos-Cas9 attP2* fly strain by Rainbow Transgenic Flies. Adult flies that were injected as embryos were crossed to second chromosome balancer stocks. Candidate progeny males from this first cross containing the *CyO* balancer chromosome were crossed to *vGlutSS1*/*CyO* virgin females. Candidate males that exhibited failed complementation were subsequently crossed to a *yw*; *20XUAS-B2*; *n-syb-GAL4* fly strain and larval progeny were screened by immunostaining. Males whose progeny exhibited positive immunostaining were subsequently crossed to a second chromosome balancer stock to establish stable lines.

### Germline excision

Germline excision of the STOP cassettes of *B2RT-STOP-B2RT-vGlut-40XV5* and *B2RT-STOP-B2RT-vGlut-40XMYC* were accomplished by crossing them to a fly strain containing the germline driver *nos-GAL4* in combination with a *20XUAS-B2* recombinase transgene. A fly strain containing all 3 genetic components was then crossed to a second chromosome balancer stock. Individual progeny males from this first cross were crossed a second time to the second chromosome balancer stock and larval progeny were screened for 40XV5 or 40XMYC protein expression to identify flies in which the STOP cassette had been excised in the germline.

### Lethal phase analysis

*B2RT-STOP-B2RT-vGlut-40XV5*/*CyO, _Dfd-YFP_* and *B2RT-STOP-B2RT-vGlut-40X-MYC*/*CyO, _Dfd-YFP_* were separately crossed to *vGlut^SS1^*/*CyO, _Dfd-YFP_*. Flies of each cross were allowed to lay eggs for 24 hours on grape juice agar plates. After aging for 24 hours post-collection, larva were scored for the presence of the Dfd-YFP marker using a Zeiss Lumar fluorescence microscope equipped with a YFP-specific filter. No non-Dfd-YFP crawling larva was observed. However, when non-Dfd-YFP embryos were dissected out of their egg cases, they contained embryos that appeared to have completed embryonic development as mouth hooks, gut, and larval cuticle were present. All experiments were carried out at 25°C.

### Immunostaining

Larval and adult immunostaining were performed as previously described (Petersen and Stowers 2011). Primary antibodies and dilution factors: The SYN (3C11) mAb 1:20 developed by E. Buchner (Klagges *et al.* 1996), Brp nc82 mAb 1:40, Dlg 4F3 mAb 1:40, and FasII 1D4 mAb 1:20 were obtained from the Developmental Studies Hybridoma Bank, created by the NICHD of the NIH and maintained at The University of Iowa, Department of Biology, Iowa City, IA 52242. rat anti-V5 SV5-P-K (Novus NBP2-81037) 1:200, rabbit anti-V5 SV5-P-K (Novus NBP2-52653) 1:200, rat anti-MYC 9E10 (Novus NBP2-81020) 1:200, rabbit anti-MYC 9E10 (Novus NBP2-52636), rat anti-FLAG (Novus NBP1-06712) 1:200, mouse anti-vGlut (Banerjee *et al*. 2021) 1:10, rat anti-mCherry 16D7 (ThermoFisher-M11217) 1:200, rabbit anti-Tdc2 pab0822-P, Covalab) 1:500. Secondary antibodies and dilution factors: Donkey anti-Rat Alexa 488 (Jackson Immunoresearch 712-546-153) 1:400, Goat anti-Rabbit Alexa 488 (Thermo-Fisher A32731) 1:400, Goat anti-Mouse Alexa 568 (Thermo Fisher A11031) 1:400, Goat anti-Mouse Alexa 647 (Thermo-Fisher A32728) 1:400, Goat anti-Rabbit Alexa 568 (Thermo Fisher A11036)1:400, Donkey anti-Rat Alexa 568 (Thermo-Fisher A78946) 1:400. Larval muscles were stained with Phalloidin iFluor405 (Abcam AB176752) 1:800. Confocal microscopy was performed using a Leica Stellaris DMI8 inverted confocal scanning light microscope in the microscopy facility at the Montana State University Center for Biofilm Engineering. For direct comparisons of signal intensity between genotypes shown in Fig. 3 and Fig. 4, gain and laser intensity were established on an initial genotype and these settings were maintained for all subsequent genotypes of the same comparison group.

## Results

### Strategy for multimer generation

The approach for generating highly multimerized epitope tags involved starting with 5X tandem copies of epitope tags commercially generated via gene synthesis with strategically placed *Asc I* and *Xho I* restriction sites upstream, and *Sal I* and *Not I* restriction sites downstream, of the 5X epitope tag sequence (Fig. 1ai, illustrated for the V5 epitope tag). This starting plasmid was cut with the restriction enzymes *Asc I*/*Xho I* to generate the vector for restriction enzyme cloning and, separately, with *Asc I*/*Sal I* to generate the insert (Fig. 1aii). The insert was restriction enzyme cloned into the vector (Fig. 1aiii) by taking advantage of the compatible cohesive ends of *Xho I* and *Sal I*. The resulting plasmid contains 10XV5 copies of the epitope tag while at the same time regenerating the initial unique restriction enzyme configuration due to the *Xho I*/*Sal I* hybrid site between the 5XV5 repeats not being recognized by either restriction enzyme. This strategy was sequentially repeated an additional 3 times with a doubling of the epitope tag copy number each round to generate 20X, 40X, and 80X copies of the epitope tag. The strategy shown for the V5 epitope tag in Fig. 1a was identical for the other 5 epitope tag multimers. The only exception was that attempts to generate an 80XHA epitope tag multimer were not successful, presumably due to sequence-specific instability.

**Fig. 2.**
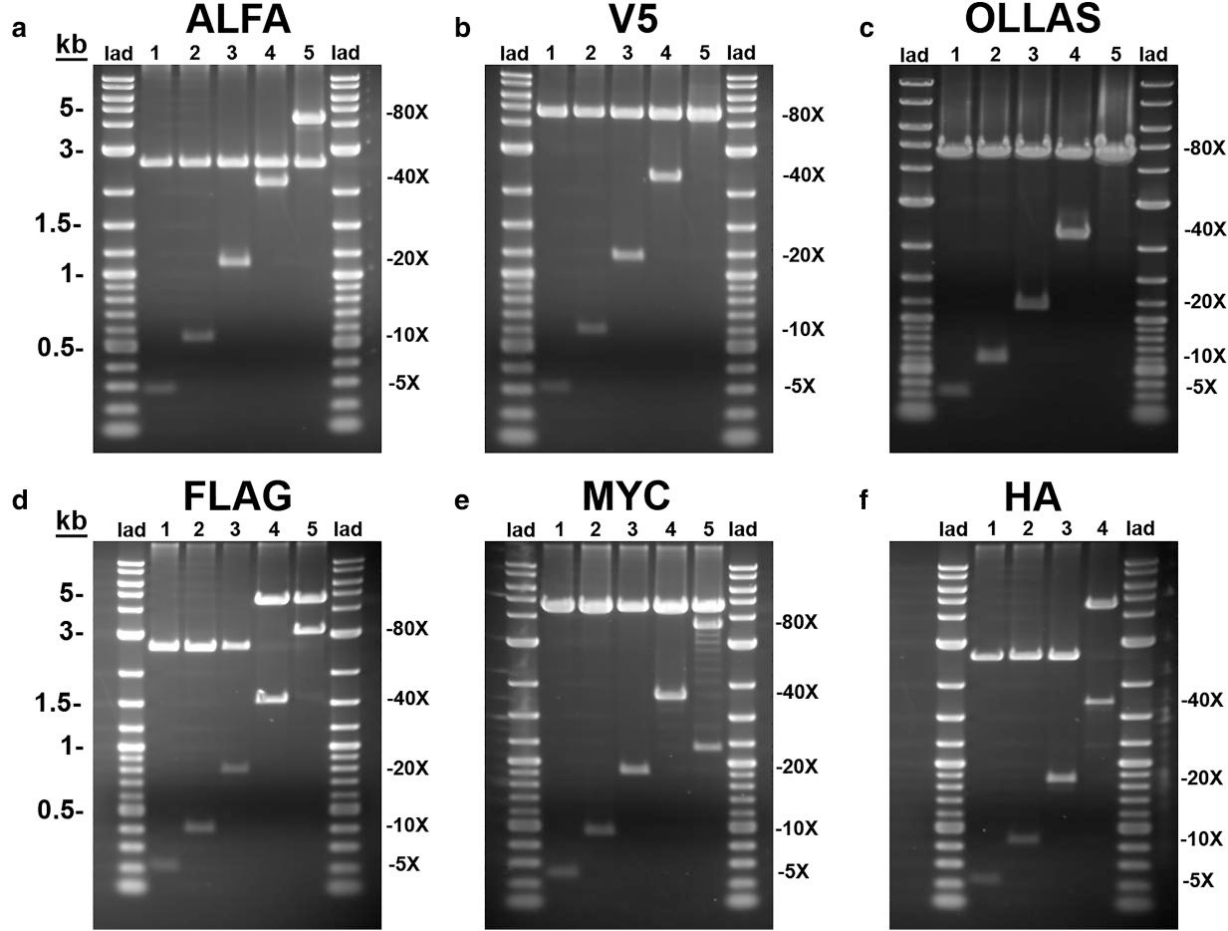
Epitope tag multimer plasmids. Ethidium bromide-stained agarose gels of *Xho I*/*Not I* restriction digests of each epitope tag multimer plasmid. a) ALFA; b) V5; c) OLLAS; d) FLAG; e) MYC; and f) HA. DNA 1 kb ladder plus fragment sizes are indicated on the left side of a) and d). The locations of the sizes of the indicated repeat multimers are indicated on the right side of each gel. The 80X MYC repeat is not entirely stable, e) lane 5. The 80V5, b) lane 5, and 80X OLLAS, c) lane 5, multimer inserts run as a doublet at a similar molecular weight as the vector. Epitope tag multimers were assembled in either the 2.6 kb *pUC57Kan* plasmid or the 4.6 kb *pUC57Kan* vGlut donor vector. Some multimer repeats were empirically determined to exhibit greater stability in the latter plasmid. Attempts to generate an 80XHA multimer were not successful.

Ethidium bromide/agarose gel electrophoresis of restriction digests of each epitope tag multimer plasmid is shown in Fig. 2. For each epitope tag multimer, a flexible GSGG linker was included between each tag to minimize the chances of the tag altering the localization or function of the protein being tagged and also maximizing the likelihood of each epitope tag being accessible to the anti-epitope tag antibody. All multimers of each of the 6 epitope tags are in the same open reading frame (Fig. 1b). This allows for a convenience of modularity such that once an initial construct has been generated containing any one of the epitope tag multimers, any other epitope tag multimer can easily be swapped in as desired in-frame in a single directional restriction enzyme cloning step. A complete list of each epitope tag multimer plasmid and its corresponding Addgene accession number is included in Supplementary Table 1.

### Demonstration of proof-of-principle in vivo

**Fig. 3.**
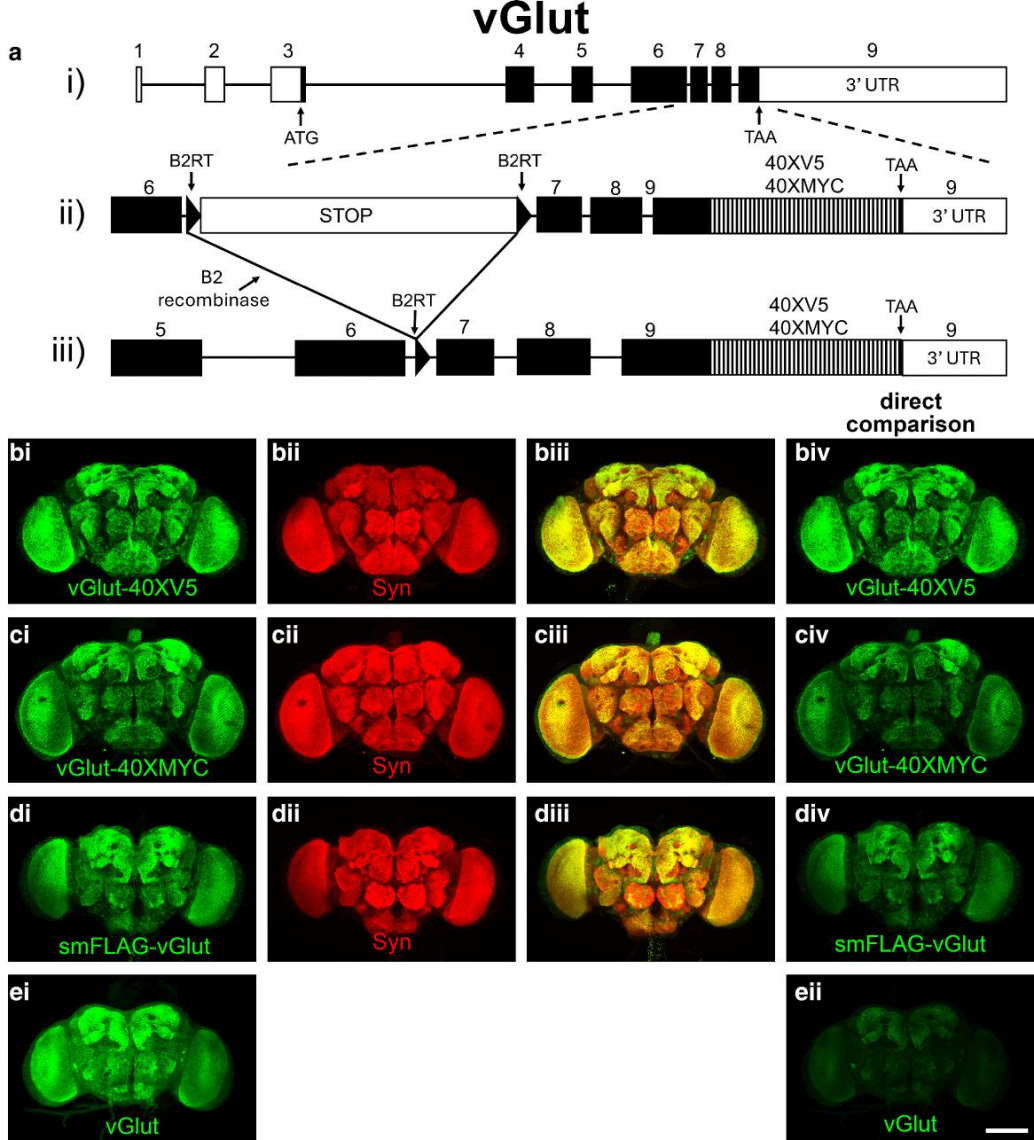
Genome editing strategy and vGlut epitope multimer-tagged expression in adult *Drosophila* brain. ai) genomic intron/exon structure of the vGlut gene with exons as rectangles and coding sequences in black. aii) the genome edits to vGlut included a STOP cassette flanked by B2 recombinase target sites (B2RTs) in the intron between exons 6 and 7 and 40 in-frame tandem repeats of either the V5 or MYC epitope tags immediately preceding the TAA stop codon. aiii) conditional expression of vGlut-40XV5 or vGlut-40XMYC results from selective expression of the B2 recombinase in neurons of interest using a GAL4 or split-GAL4 driver in combination with a *UAS-B2* recombinase transgene. bi) vGlut-40XV5; bii) Synapsin; biii) overlay; biv) vGlut-40XV5; ci) vGlut-40XMYC; cii) Synapsin; ciii) overlay; civ) vGlut-40XMYC; di) smFLAG-vGlut; dii) Synapsin; diii) overlay; div) smFLAG-vGlut; ei) vGlut; eii) vGlut. Bi, Ci, Di, and Ei images were adjusted post-acquisition to comparable levels of intensity. biv, civ, div, and eii were acquired and processed identically to reveal the relative differences in signal intensity. Scale bar: 100 μm. Complete genotype: b) *yw*; *B2RT-vGlut-40XV5 GE*/*+*; c) *yw*; *B2RT-vGlut-40XMYC GE*/*+*; d) yw; *smFLAG-vGlut GE*/*+*; e) *yw*.

As proof-of-principle of the in vivo utility of the multimerized epitope tags, the 40XV5 and 40XMYC multimers were genome edited into the Drosophila vesicular glutamate transporter gene vGlut (Fig. 3a). The genome editing strategy for vGlut included separate fusion of either a 40XV5 or 40XMYC epitope tag onto the carboxy-terminus and a STOP cassette (Fisher *et al.* 2017) flanked by B2 recombinase target sites (*B2RTs*) inserted in the intron between exons 6 and 7 for conditional expression (Fig. 3aii) to create *B2RT-STOP-B2RT-vGlut-40XV5* and *B2RT-STOP-B2RT-vGlut-40XMYC*. Upon selective expression of the B2 recombinase using a GAL4 or split-GAL4 driver in combination with a *20XUAS-B2* recombinase transgene, the STOP cassette is excised in glutamatergic neurons of interest and allows vGlut-40XV5 or vGlut-40XMYC expression (Fig. 3aiii). Epitope tagging at the native genomic location ensures endogenous expression levels, thereby obviating artifactual mis-localization that can result from ectopic overexpression using binary transcription systems such as GAL4/UAS.

After successful genome-editing, germline excisions of the STOP cassettes of both *B2RT-STOP-B2RT-vGlut-40XV5* and *B2RT-STOP-B2RT-vGlut-40XMYC* were generated to create *B2RT-vGlut-40XV5-GE* and *B2RT-vGlut-40XMYC-GE.* In these fly strains, vGlut-40XV5 and vGlut-40XMYC should be expressed in all glutamatergic neurons since the STOP cassette blocking their expression has been excised. The expression patterns of vGlut-40XV5 (Fig. 3bi) and vGlut-40XMYC (Fig. 3ci) localize to the synaptic neuropil in adult brain, highly similar to a previously reported smFLAG-vGlut (Certel *et al.* 2022) (Fig. 3di), as well as endogenous vGlut (Fig. 3ei). These patterns of expression are also similar to that of the pan-synaptic vesicle marker Synapsin (Figs. 3bii, Cii, and Dii), but not identical, as Synapsins are thought to localize to all synaptic vesicles independent of neurotransmitter usage (Fdez and Hilfiker 2006; Cesca *et al.* 2010).

To evaluate the relative detection sensitivity in the adult brain of vGlut-40XV5 and vGlut-40XMYC compared to smFLAG-vGlut and endogenous vGlut, the same images of the brains shown in Figs. 3bi, Ci, Di, and Ei, which were acquired using identical settings and enhanced post-acquisition such that the signal intensities were at comparable levels, are shown in Figs. 3 Biv, Civ, Div, and Eii under identical acquisition and processing conditions. VGlut-40XV5 exhibits the strongest intensity, followed by vGlut-40XMYC and smFLAG-vGlut which are similar, with endogenous vGlut exhibiting the lowest signal intensity. A caveat to this comparison is the epitope tags for each vGlut epitope tag fusion protein are different and the antibodies recognizing them will have different binding affinities such that the observed signal intensities may not be a direct reflection of the quantity of the different numbers of epitope tags.

**Fig. 4.**
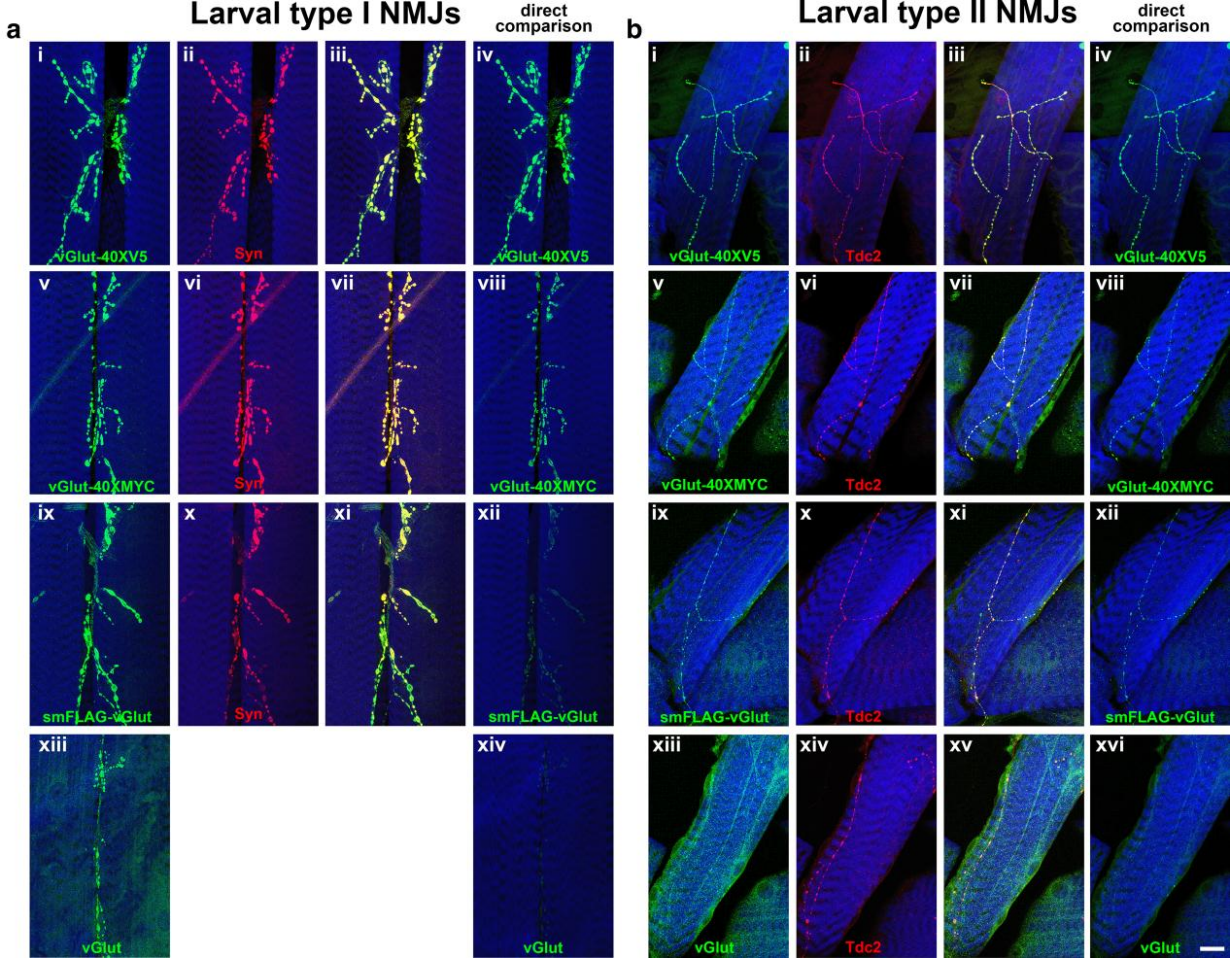
vGlut epitope multimer expression at the type I and type II neuromuscular junction of third instar larval *Drosophila*. a) larval type I NMJs at muscle 6/7 boundary. ai) vGlut-40XV5; aii) Synapsin; aiii) overlay; aiv) vGlut-40XV5; av) vGlut-40XMYC; avi) Synapsin; avii) overlay; aviii) vGlut-40XMYC; aix) smFLAG-vGlut; ax) Synapsin; axii) smFLAG-vGlut; axiii) vGlut; axiv) vGlut. Images shown in ai), av), aix), and axiii) were processed post-acquisition to comparable levels of intensity. aiv), aviii), axii), and axiv) were imaged and processed identically to reveal the relative differences in signal intensity. b) larval type II NMJs at muscle 5. bi) vGlut-40XV5; bii) Tdc2; biii) overlay; biv) vGlut-40XV5; bv) vGlut-40XMYC; bvi) Tdc2; bvii) overlay; bviii) vGlut-40XMYC; bix) smFLAG-vGlut; bx) Tdc2; bxi) overlay; bxii) smFLAG-vGlut; bxiii) vGlut; bxiv) Tdc2; bxv) overlay; bxvi) vGlut. Images in bi), bv), bix), an bxiii) were processed post-acquisition to similar levels of signal intensity (to the extent possible). Images shown in biv), bviii), bxii), and bxvi) were acquired and processed identically to reveal the relative differences in signal intensity. Scale bar: 20μm. Complete genotype: ai-iv) yw; B2RT-vGlut-40XV5 GE/+; av-viii) yw; B2RT-vGlut-40XMYC GE/+; aix-xii) yw; smFLAG-vGlut GE/+; axiii, xiv) yw. bi-iv) yw; B2RT-vGlut-40XV5 GE/+; bv-viii) yw; B2RT-vGlut-40XMYC GE/+; bix-xii) yw; smFLAG-vGlut GE/+; bxiii, xiv) yw.

A similar assessment of vGlut-40XV5 and vGlut-40XMYC protein localization was performed at both the type I and type II Drosophila larval neuromuscular junctions (NMJs), which are both known to be glutamatergic (Johansen *et al.* 1989). As expected, vGlut-40XV5 and vGlut-40XMYC localize to the synaptic terminals of both type I and type II NMJs (vGlut-40XV5, Figs. 4ai and 4bi, respectively; vGlut-40XMYC, Figs. 4av and 4bv, respectively), similar to smFLAG-vGlut (Figs. 4aix and 4bix, respectively). Endogenous vGlut is also detected at larval type I NMJs (Fig. 4axiii) but is only borderline detectable at type II NMJs (Fig. 4bxiii). The larval type I NMJ immunonstains were also co-labeled for the pan-SV protein Synapsin and the expression patterns of all 3 vGlut epitope tag fusion proteins exhibit nearly precise overlap (Figs. 4aiii, 4avii, and 4axi).

To gauge the relative detection sensitivity at the larval type I NMJs of vGlut-40XV5 and vGlut-40XMYC compared to smFLAG-vGlut and endogenous vGlut, the same images of the NMJs shown in Figs. 4ai, Av, Aix, and Axiii, which were acquired using identical settings and enhanced post-acquisition such that the signal intensities were at comparable levels, are shown in Figs. 4aiv, 4aviii, 4xii, and 4xiv under identical acquisition and processing conditions. The order of signal intensity strength is vGlut-40XV5 > vGlut-40XMYC > smFLAG-vGlut > vGlut. The same order of signal intensity strength was observed at larval type II NMJs (identified by the expression of the tyramine/octopamine neurotransmitter synthesis enzyme Tdc2) when the images shown in Figs. 4bi, Bv, Bix, and Bxiii were acquired and processed identically with vGlut-40XV5 being strongest (Fig. 4biv), followed by vGlut-40XMYC (Fig. 4bviii), then smFLAG-vGlut (Fig. 4bxii), and lastly endogenous vGlut (Fig. 4bxvi).

### Assessment of conditionality of expression

To determine if vGlut-40XV5 and VGLUT-40XMYC expression requires excision of the STOP cassette, their expression was compared in the presence and absence of the STOP cassette in adult brain and at the larval neuromuscular junction. In the absence of the STOP cassette in the germline excision variants, vGlut-40XV5 and vGlut-40XMYC are strongly expressed in adult brain (Supplementary Fig. 1, b and h, respectively). In the presence of the STOP cassette, no expression of either vGlut-40XV5 or vGlut 40XMYC was observed (Supplementary Fig. 1, e and k, respectively). Similar results were observed at the larval NMJ. In the absence of the STOP cassette in the germline excision variants, vGlut-40XV5 and vGlut-40XMYC are strongly expressed at the larval NMJ (Supplementary Fig. 2, b and h, respectively). In the presence of the STOP cassette, no expression of either vGlut-40XV5 or vGlut 40XMYC was observed (Supplementary Fig. 2, e and k, respectively). These results demonstrate the STOP cassette is highly effective in preventing leaky expression and thereby that the expression of vGlut-40XV5 and vGlut-40XMYC is conditional, as intended by design.

### Characterization of vGlut function pre- and post-excision of the STOP cassette

Given the demonstrated lack of expression of vGlut-40XV5 and vGlut-40XMYC in *B2RT-STOP-B2RT-vGlut-40XV5* and *B2RT-STOP-B2RT-vGlut-40XMYC*, respectively, it would be predicted that both are complete loss-of-function alleles of vGlut and thus they should exhibit lethality at the late embryonic stage of development as has previously been determined for vGlut null alleles (Daniels *et al.* 2006). To determine if this is the case, the *B2RT-STOP-B2RT-vGlut-40XV5* and *B2RT-STOP-B2RT-vGlut-40XMYC* chromosomes were placed in heterozygous combination with the established *vGlut^SS1^* null allele. As predicted, no first instar larva emerged from their egg cases in either heterozygous combination. Dissection of the heterozygous embryos aged for 24 hours after egg-laying revealed larva complete with mouth hooks, gut, and larval cuticle. These results thus indicate a late embryonic lethal phase, the same as has been previously reported for *vGlut* null alleles. These results are consistent with the idea that *B2RT-STOP-B2RT-vGlut-40XV5* and *B2RT-STOP-B2RT-vGlut-40XMYC* are complete loss-of-function *vGlut* alleles.

In contrast, germline excision variants of both vGlut-40XV5 and vGlut-40XMYC, in which the STOP cassette has been excised, are homozygous viable, fertile, and in no obvious way distinguishable from *yw* controls. To assess whether fusion of the 40XV5 or 40XMYC epitope tag multimers to the carboxy-terminus of vGlut cause any obvious morphological defects of the nervous system, homozygous *vGlut-40XV5* and *vGlut-40XMYC* larva with a germline excision of the STOP cassette were subjected to immunostaining using various neuronal markers at the larval NMJ and larval ventral nerve cord. Immunostaining of the Brp active zone protein at the larval NMJ in both the *vGlut-40XV5* germline excision (Supplementary Fig. 3b) and *vGlut-40XMYC* germline excision (Supplementary Fig. 3c) was not obviously different from a *yw* control (Supplementary Fig. 3a). Similarly, immunostaining of the Dlg post-synaptic scaffolding protein at the larval NMJ in the *vGlut-40XV5* germline excision (Supplementary Fig. 3e) and *vGlut-40XMYC* germline excision (Supplementary Fig. 3f) was not noticeably different from a *yw* control (Supplementary Fig. 3d). No discernable defects in neuronal morphology were observed in immunostains of the larval ventral nerve cord using either the tyrosine decarboxylase 2 (vGlut-40XV5-Supplementary Fig. 3h; vGlut-40XMYC-Supplementary Fig. 3i; yw-Supplementary Fig. 3g) or FasII (vGlut-40XV5-Supplementary Fig. 3k; vGlut-40XMYC-Supplementary Fig. 3l; yw-Supplementary Fig. 3j) neuronal markers that allow visualization of distinct subsets of individual neurons. These observations on viability, fertility, and neuronal morphology, taken together with the immunostaining data in both the adult brain and larval type I and type II NMJs demonstrating presynaptic vGlut-40XV5 and vGlut-40XMYC localization similar to endogenous vGlut and the pan-SV protein Synapsin, suggest the 40XV5 and 40XMYC epitope tag multimers fused onto the vGlut carboxy-terminus have little, if any, effect on vGlut protein localization or function.

### Conditional expression in individual neurons

**Fig. 5.**
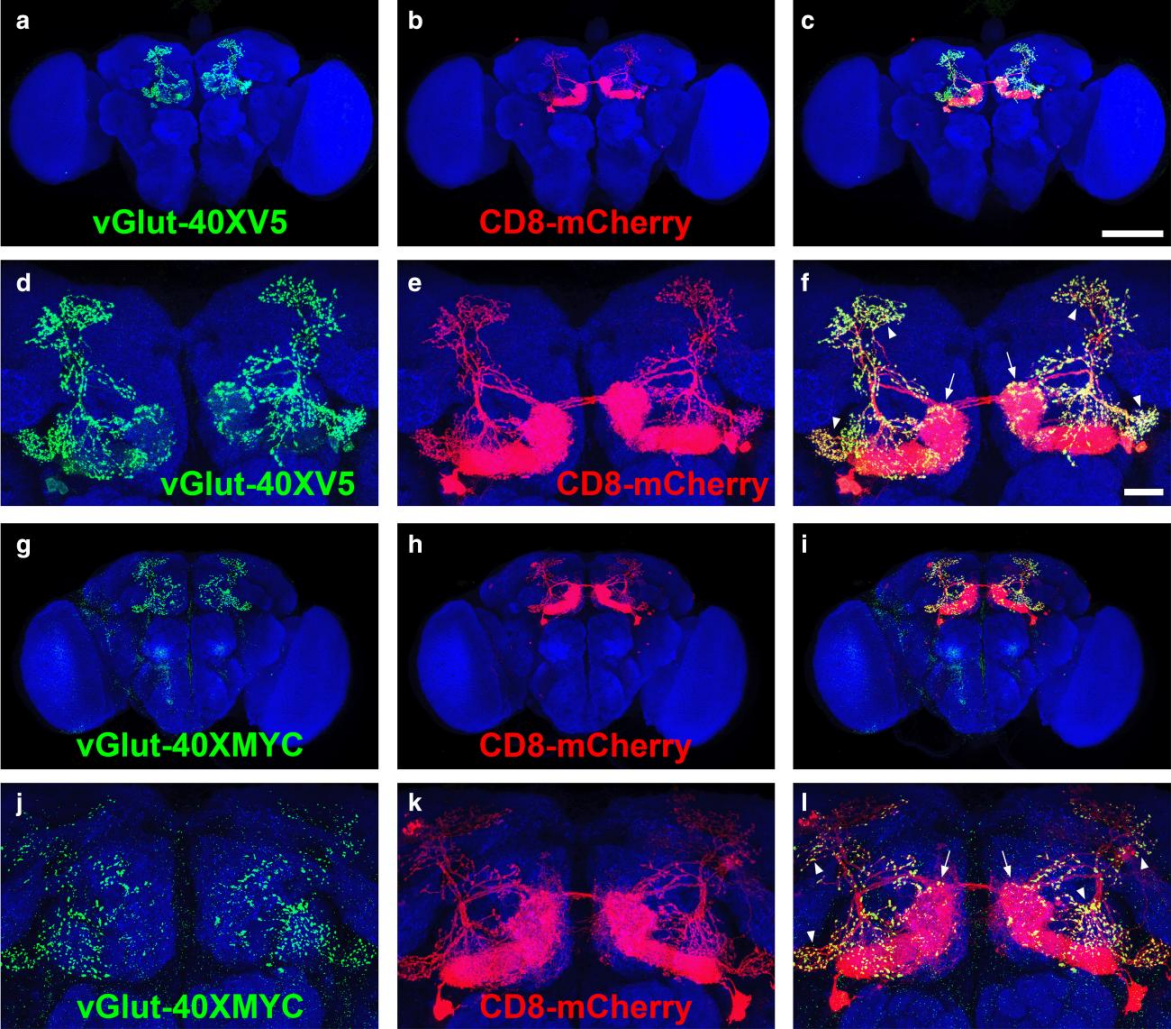
Conditional expression of vGlut-40XV5 and vGlut-40XMYC in Drosophila MBON-5/6 adult brain neurons. a-c) whole brain images. a) vGlut-40XV5; b) CD8-mCherry; c) overlay; d-f) high resolution images. d) vGlut-40XV5; e) CD8-mCherry; f) overlay. g-i) whole brain images. g) vGlut-40XMYC; h) CD8-mCherry; i) overlay. j-l) high resolution images. j) vGlut-40XMYC; k) CD8-mCherry; l) overlay. Both vGlut-40XV5 and vGlut-40XMYC localize to pre-synaptic terminals of MBON 5/6 neurons. Arrows indicate dendritic regions. Arrowheads indicate presynaptic terminals. Scale bars: a-c), g-i) 100 μm; d-e), j-l) 20 μm. Complete genotype: a-f) yw; B2RT-STOP-B2RT-vGlut-40XV5/R30E08-p65.AD; UAS-CD8-mCherry, 20XUAS-B2/R53C10-GAL4.DBD; g-l) yw; B2RT-STOP-B2RT-vGlut-40XMYC/R30E08-p65.AD; UAS-CD8-mCherry, 20XUAS-B2/R53C10-GAL4.DBD.

To visualize expression of vGlut-40XV5 and vGlut-40XMYC in neuronal subsets of the adult Drosophila brain, each were separately expressed in the polarized glutamatergic mushroom body output neurons 5 and 6 (MBON 5/6) using the MBON-5/6-specific driver *MB434B* to selectively express the B2 recombinase using a *20XUAS-B2* recombinase transgene. In these flies, the STOP cassette is excised specifically in MBON-5/6 neurons, thereby relieving the STOP cassette suppression of vGlut-40XV5 and vGlut-40XMYC expression. As expected, in whole brain images, vGlut40XV5 expression (Fig. 5a) was observed exclusively in MBON5/6 neurons visualized with the plasma membrane fluorescent reporter CD8-mCherry (Fig. 5b). Higher resolution images of vGlut-40XV5 (Fig. 5d) in MBON-5/6 neurons (Fig. 5e) reveals an asymmetric distribution of vGlut-40XV5 such that it is absent from the dendritic regions (arrows, Fig. 5f) but specifically localizes to presynaptic terminals (arrowheads, Fig. 5f), as expected for a synaptic vesicle protein.

Similar results were obtained for vGlut-40XMYC expression in MBON-5/6 neurons. In whole brain images, vGlut-40XMYC was observed (Fig. 5g) exclusively in MBON-5/6 neurons (Fig. 5h). Higher resolution images of vGlut-40XMYC (Fig. 5j) in MBON-5/6 neurons (Fig. 5k) reveals vGlut-40XMYC does not localize to dendritic regions (arrows, Fig. 5l, but exclusively localizes to presynaptic regions (arrowheads, Fig. 5l).

## Discussion

A detailed knowledge of protein localization within a cell is critical to understanding its function, but the ability to detect expression can be challenging for low abundance proteins. To overcome this challenge, a set of 29 plasmid DNA clones was developed that encodes multimers of 6 common epitope tags with repeat numbers ranging from 5X to 80X using a reiterative strategy that doubled the repeat number with each round of cloning. This cloning strategy should be also generally applicable for multimerization of other types of DNA sequences for different research purposes and could include enhancer sequences, fluorescent proteins, etc.

As proof-of-principle of the in vivo utility of the epitope tag multimers, the 40XV5 and 40XMYC epitope tags were fused to the carboxy-terminus of *Drosophila* vGlut via genome-editing. Both proteins exhibited robust, easily detectable expression in the *Drosophila* adult brain and larval neuromuscular junction, even in individual neurons. Neither protein appeared to be negatively affected by addition of the 40X multimers to the vGlut carboxy-termini with regard to subcellular localization or function based on their localization to pre-synaptic terminals, absence of defects on neuronal morphology, viability or fertility in homozygous animals in which the STOP cassette has been excised. As expected, both vGlut-40XV5 and vGlut-40XMYC exhibited higher signal intensity as compared to a previously described smFLAG-vGlut, presumably due to the higher number of epitope tag repeats (40X vs 10X, respectively), although the comparison is imperfect since different primary anti-epitope antibodies were necessarily used for the different vGlut epitope-tagged proteins. These successes with the 40XV5 and 40XMYC epitope tag multimers imply that the other epitope tag multimers will also be functional since they were all generated using the identical strategy. However, whether any given protein will tolerate multimerized epitope tagging at a specific location can only be determined empirically.

The epitope tag multimers described herein have several potential advantages over the spaghetti monster proteins that encode 10X copies of epitope tags and have heretofore served as the standard for tagging proteins of interest with multiple copies of epitope tags. First and foremost is the epitope tag multimers potentially offer up to an 8-fold enhancement in sensitivity detection (80X vs 10X). For low abundance proteins this sensitivity difference could be essential for the success of experimental objectives but even for higher abundance proteins enhanced detection sensitivity is beneficial. A second advantage is these multimerized epitope tags offer a range of multimer repeat number. This makes them potentially useful for a variety of applications such that lower repeat numbers may be more appropriate for some applications while higher repeat numbers may be more suitable for other applications. A third advantage is the epitope tag multimer sequences are encodable in smaller DNA sequences than the spaghetti monster proteins as the 10X multimers presented here are encoded in approximately half the number of nucleotides as the spaghetti monster multimers containing 10X epitope tag repeats. Cloning and downstream applications such as genome editing will thus be more efficient with smaller DNA sequences. It is also worth noting that 20X multimers are in the same size range of amino acids as GFP and 40X multimers are in the same size range at tdTomato, 2 proteins commonly used for fluorescent tagging. Thus, for the 40X multimers and below, these epitope tags are in the same size range as routinely utilized fluorescent tags (Avellaneda and Schnorrer 2022). A fourth potential advantage is they are less likely to disrupt protein function. Between each epitope tag repeat, there is a small 4 amino acid linker containing a GSGG sequence that provides more flexibility than the spaghetti monster proteins containing fluorescent proteins of rigid 3-dimensional structure. The flexible linkers may be particularly helpful when it is desirable to place an epitope tag multimer internally where insertion of a larger, less flexible fluorescent protein would be more likely to disrupt function.

The epitope tag multimers described herein are potentially useful for a wide range of research applications that involve the use of antibodies to detect proteins including immunostaining, biochemistry, Western blots, ELISA, flow cytometry, and expansion microscopy, among others, where enhanced detection sensitivity would be advantageous. These multimers might be especially beneficial for expansion microscopy since the intensity of the immunofluorescent signal diffuses with the third power of the expansion factor due to isotropic 3-dimensional expansion. Currently, for expansion microscopy it is possible to achieve at least 10-fold expansion with a single round of expansion (Damstra *et al.* 2023) and 40-fold expansion with reiterative expansion (Tian *et al.* 2024). Fluorescence signal is thus diluted 1000-fold and 64,000-fold for 10X and 40X expansion, respectively. Many proteins will not have expression levels and available high-affinity antibodies that will allow detection at these dilution factors without signal enhancement. Lastly, although proof-of-principle of the epitope tag multimers was demonstrated in the *Drosophila* model system, they are potentially applicable to any genetically manipulable species since the genetic code is universal.

## Supplementary Material

jkaf070_Supplementary_Data

## Data Availability

The complete sequences of the *B2RT-STOP-B2RT-vGlut-40XV5* and *B2RT-STOP-B2RT-vGlut-40XMYC* donor plasmids are included as Supplementary Figs. 4a, 4b, 5a, and 5b. The guide RNA and donor plasmids will be made available upon request. All epitope tag multimer plasmids have been submitted to Addgene for distribution. A list of the epitope tag plasmids and their Addgene accession numbers are included in Supplementary Table 1. Fly strains original to this publication will be deposited at the Bloomington Drosophila Stock Center or made available upon request. Supplementary Figs. 1-3 are available on GSA FigShare: https://doi.org/10.25387/g3.28685285. Supplemental material available at G3 online.
