# Supplementary material for: Multimerized epitope tags for high-sensitivity protein detection
Source: G3 (Bethesda). 2025 Apr 7;15(6):jkaf070. doi: 10.1093/g3journal/jkaf070 (PMC12134993; doi:10.1093/g3journal/jkaf070)
Supplement: jkaf070_Supplementary_Data [file jkaf070_supplementary_data.zip › FigureS4A_G3-2025-405811.docx]

**B2RT-STOP-B2RT-vGlut-40XV5**

tcgcgcgtttcggtgatgacggtgaaaacctctgacacatgcagctcccggagacggtcacagcttgtctgtaagcggatgccgggagcagacaagcccgtcagggcgcgtcagcgggtgttggcgggtgtcggggctggcttaactatgcggcatcagagcagattgtactgagagtgcaccagatgcggtgtgaaataccgcacagatgcgtaaggagaaaataccgcatcaggcgccattcgccattcaggctgcgcaactgttgggaagggcgatcggtgcgggcctcttcgctattacgccagctggcgaaagggggatgtgctgcaaggcgattaagttgggtaacgccagggttttcccagtcacgacgttgtaaaacgacggccagtgaattcAAAAGTCATACATACACAAAAGGACACGCACCCACACAGACCCATCATAGAAACCAATTTGAAATTCTGAATCCTGCTTGACGCTCGTTAATAAATGCCTGGAGCAGTTTTTAATGATTGCTGAGCTTCTGTCACTCATCCCGTAAAATGTGTTTCCGGATTTTTGCAGACTGTGTTCATGAACTGGACGGTCGCCGTGGAGAGCCATGTGGACTCGTCCTTCTTCTGGGGCTATCTGGTGACGCAGATTCCCGGCGGCTTCATCGCCTCCAAGTTCCCGGCCAACAAGATATTCGGACTGTCCATCGTGAGCTCCGCCACGCTGCATCTCTTCGTGCCATTCGCCATGACCCTGATGCACGGTCATGTGGTGATTTGCGTGAGGGTCCTGCAAGGACTCTTCGAGGTATGTAGCCCAACCAACCTAACAACTTGCCCAATTCGATTTTGCCACAGATCAAAGCCGGGGATTACGGCTGCGTGATGTTGCGAAAATGGGGAGGGTTAGATTTTGCTTTATGATAAAGCAAATATGTGAAATGGAAACATGTCCTAAGCCCTCAAAGGGATGGGGGTCCTGGGAGGGCAGGAAAAAATAACAAAAGCATTTATTTTAATGCGATTTCTTTGTGCCAAGGACTGGGAAGTATGTCATTTTTCTTCGAATCGGAAGGGAGAGAATGATACTTTGTAAAAAGGATGTTTGTCTATTCGAGTTCCGAAGTAACTATATTTTAGATATATATTTTTAATTTCACAAAGCACATTTTCTTAAGATACTATAACTGCATGAGACTAAGTGCAgagtttcattaaggaataactaattccctaatgaaactcgcgtcgcgccacttcaacgctcgatgggagcgtcagcgtcgcgccacttcaacgctcgatgggagcgtcattggtgggcggggtaaccgtcgaaatcagtgtttacgcttccaatcgcaacaaaaaattcactgcaacactgaaaagcatacgaaaacgatgaagattgtacgagaaaccataaagtattttatccacaaagacacgtatagcagaaaagccaagttaactcggcgataagttgtgtacacaagaataaaatcggccagattcagtgttgtcagaaataagaaaaccccactatgtttttctttgccttttctttctcccagcgatcattcatttcgtggtgaaagaacggggtcattgcacggagtttcgactgcgggaaagcagagctgccgttcacttcgtctataattagcgctttctattttccccgattcgggccgctgctgcgcttttccgcctgctgtttgtggcaagtgtagcagcaggctgtgcacgcagtgtggcatgcacttggctttccaccgttggtatcgattctctgggacgatgagtcattcctttcggggccacagcataatcgttgccagctcaccgaaatggtgacttcatttcttaactgccgtcaagcatgcgattgtacatacatacatatttatatatgtacatatttatgtgactatggtaggtcgatataatagcaatcaacgcaagcaaatgtgtcagtcctgcttacaggaacgattctatttagtaattttcgttgtataaagtaattatgtatgtatgtaagccccataaatctgaaaaccggtactagtacgaaagtcgatccaacatggcgacttgtcccatccccggcatgtttaaatatactaattattcttgaactaattttaatcaaccgatttatctctcttccgcaggtctaactaactaacaaaattgactaagtgagatctttgtgaaggaaccttacttctgtggtgtgacataattggacaaactacctacagagatttaaagctctaaggtaaatataaaatttttaagtgtataatgtgttaaactactgattctaattgtttgtgtattttagattccaacctatggaactgatgaatgggagcagtggtggaatgcctttaatgaggaaaacctgttttgctcagaagaaatgccatctagtgatgatgaggctactgctgactctcaacattctactcctccaaaaaagaagagaaaggtagaagaccccaaggactttccttcagaattgctaagttttttgagtcatgctgtgtttagtaatagaactcttgcttgctttgctatttacaccacaaaggaaaaagctgcactgctatacaagaaaattatggaaaaatatttgatgtatagtgccttgactagagatcataatcagccataccacatttgtagaggttttacttgctttaaaaaacctcccacacctccccctgaactcgaaacataaaatgaatgcaattgttgttgttaacttgtttattgcagcttataatggttacaaataaagcaatagcatcacaaatttcacaaataaagcatttttttcactgcattctagttgtggtttgtccaaactcatcaatgtatcttatcatgtctggatcccaaactcatcaatgtatcttatcatgtctggatcgagtttcattaaggaataactaattccctaatgaaactcTTGGAATATAGGACAATATCCTAATTGACTTATTTAAATAATCCATTTATTTAAACATAATCTCTTTTAGGGCGTTACCTATCCAGCTTGCCATGGTATCTGGCGTTTCTGGGCGCCGCCCATGGAGCGCTCCCGACTGGCGACGCTGGCCTTTTCCGGTTCCTATGCGGGCGTGGTGGTTGGACTTCCGCTCTCCGGACTTCTGGCCGATGCCGTGGGCTACCAGGCGCCGTTCTACGCCTACGGGGTGTTCGGAATCATATGGTACATGTTCTGGATATGGTTGTGCTTCGAGAACCCGCGCAAACATCCGGCCATCAGCATACCCGAGTTGAAGTACATCGAGAAATCGCTCGGGGAGTCGGCTCATCCCACGATGCCATCCCTGAAGACGACTCCGTGGCGGGAAATGATGCGCTCGATGCCGGTCTACGCCATCATTGTGGCCAACTTCTGCCGCTCCTGGAACTTCTACCTCCTGGTGCTGTTCCAGTCCTCGTTCCTCAAGCACAAGTTCGGTTTTAAGGTGGAGGAGGCGGGCTTCGTGGGCTCGCTGCCCCACTTGATCATGACTACGATAGTTCCATTTGGCGGCATGTTGGCGGATCACCTGCGAAAGAATGGTATCCTGTCCACCACCAATGTGCGCAAGCTCTTCAATTGCGGCGGCTTTGGCATGGAGGGTCTGTTTTTCCTATTCGTGGCACATTCCTCAACGGCGGTAAGTTATAAACAAATTCCTTTATACTCAATACTATAAGTTGTTTTTTCCAGACGGGTGCCATGTTTGCCTTGACCTGCGGCGTGGCCTTCAGTGGCTTTGCCATATCCGGTTATAATGTCAATCACCTGGATATTGCTCCTCGTTATGCTAGTATATTGATGGGTCTTTCGAATGGAATTGGTACTCTGGCCGGCATCATTGTGCCCTATGCCCTTGATGGCCTCATCCAAGCTAATGTAAGTTGGGGTTTTTTAGTGTAAGGTAGTTTCTGTAGCTAAGGTATTCATTTGGTATTGTAGCCTACCGGGTGTTGGACTACAGTCTTCACCCTGGCCGCCTGTGTTCATTTGGTTGGCTGCACTTTCTATGGTATTTTCGCATCTGGAGAGCTGCAGCCGTGGGCGGAACCTCCGGCCGAGGAGCAAAAGGTGTGGGCTCCACCACCAGGTGCCATTACCAACACGGATCCTAGCCAGGCGGGCATGTTGGGCGACTACATGAAGGAAACCTCATTCGTAAGTTTTAAAGTTATTACGAATTATATATTAATATTTCTATCAATCGAGCCACTACCTACAAAGCGTGTTTGTGTGTTTTTTTTTGTGAATAGGGTGCCCCCGAGTACACTGAGCAGAGCCAAATGCAGCAGTCGACTGCCATTAGCTACGGCGCCACAGGACACGTGGCCAACAATCCCTTCGCCATGGCCAGTGGTGCTCCGCCCATTGCGGAGGAGGATGCCCCACCGACCTACGGGGATGTTACCAATCCTGGGCAGTATGGCTACACGCAAGGACAAATGCCGTCCTACGATCCGCAGGGATACCAGCAGCAGGGCTCCGGCGGCtggcgcgccCTTAAGCTCGAGGGCTCCGGCGGCGGCAAGCCCATCCCCAACCCCTTACTGGGCCTGGATTCCACCGGCTCCGGCGGCGGCAAGCCCATCCCCAACCCCTTACTGGGCCTGGATTCCACCGGCTCCGGCGGCGGCAAGCCCATCCCCAACCCCTTACTGGGCCTGGATTCCACCGGCTCCGGCGGCGGCAAGCCCATCCCCAACCCCTTACTGGGCCTGGATTCCACCGGCTCCGGCGGCGGCAAGCCCATCCCCAACCCCTTACTGGGCCTGGATTCCACCGGCTCCGGCGGCGTCGAGGGCTCCGGCGGCGGCAAGCCCATCCCCAACCCCTTACTGGGCCTGGATTCCACCGGCTCCGGCGGCGGCAAGCCCATCCCCAACCCCTTACTGGGCCTGGATTCCACCGGCTCCGGCGGCGGCAAGCCCATCCCCAACCCCTTACTGGGCCTGGATTCCACCGGCTCCGGCGGCGGCAAGCCCATCCCCAACCCCTTACTGGGCCTGGATTCCACCGGCTCCGGCGGCGGCAAGCCCATCCCCAACCCCTTACTGGGCCTGGATTCCACCGGCTCCGGCGGCGTCGAGGGCTCCGGCGGCGGCAAGCCCATCCCCAACCCCTTACTGGGCCTGGATTCCACCGGCTCCGGCGGCGGCAAGCCCATCCCCAACCCCTTACTGGGCCTGGATTCCACCGGCTCCGGCGGCGGCAAGCCCATCCCCAACCCCTTACTGGGCCTGGATTCCACCGGCTCCGGCGGCGGCAAGCCCATCCCCAACCCCTTACTGGGCCTGGATTCCACCGGCTCCGGCGGCGGCAAGCCCATCCCCAACCCCTTACTGGGCCTGGATTCCACCGGCTCCGGCGGCGTCGAGGGCTCCGGCGGCGGCAAGCCCATCCCCAACCCCTTACTGGGCCTGGATTCCACCGGCTCCGGCGGCGGCAAGCCCATCCCCAACCCCTTACTGGGCCTGGATTCCACCGGCTCCGGCGGCGGCAAGCCCATCCCCAACCCCTTACTGGGCCTGGATTCCACCGGCTCCGGCGGCGGCAAGCCCATCCCCAACCCCTTACTGGGCCTGGATTCCACCGGCTCCGGCGGCGGCAAGCCCATCCCCAACCCCTTACTGGGCCTGGATTCCACCGGCTCCGGCGGCGTCGAGGGCTCCGGCGGCGGCAAGCCCATCCCCAACCCCTTACTGGGCCTGGATTCCACCGGCTCCGGCGGCGGCAAGCCCATCCCCAACCCCTTACTGGGCCTGGATTCCACCGGCTCCGGCGGCGGCAAGCCCATCCCCAACCCCTTACTGGGCCTGGATTCCACCGGCTCCGGCGGCGGCAAGCCCATCCCCAACCCCTTACTGGGCCTGGATTCCACCGGCTCCGGCGGCGGCAAGCCCATCCCCAACCCCTTACTGGGCCTGGATTCCACCGGCTCCGGCGGCGTCGAGGGCTCCGGCGGCGGCAAGCCCATCCCCAACCCCTTACTGGGCCTGGATTCCACCGGCTCCGGCGGCGGCAAGCCCATCCCCAACCCCTTACTGGGCCTGGATTCCACCGGCTCCGGCGGCGGCAAGCCCATCCCCAACCCCTTACTGGGCCTGGATTCCACCGGCTCCGGCGGCGGCAAGCCCATCCCCAACCCCTTACTGGGCCTGGATTCCACCGGCTCCGGCGGCGGCAAGCCCATCCCCAACCCCTTACTGGGCCTGGATTCCACCGGCTCCGGCGGCGTCGAGGGCTCCGGCGGCGGCAAGCCCATCCCCAACCCCTTACTGGGCCTGGATTCCACCGGCTCCGGCGGCGGCAAGCCCATCCCCAACCCCTTACTGGGCCTGGATTCCACCGGCTCCGGCGGCGGCAAGCCCATCCCCAACCCCTTACTGGGCCTGGATTCCACCGGCTCCGGCGGCGGCAAGCCCATCCCCAACCCCTTACTGGGCCTGGATTCCACCGGCTCCGGCGGCGGCAAGCCCATCCCCAACCCCTTACTGGGCCTGGATTCCACCGGCTCCGGCGGCGTCGAGGGCTCCGGCGGCGGCAAGCCCATCCCCAACCCCTTACTGGGCCTGGATTCCACCGGCTCCGGCGGCGGCAAGCCCATCCCCAACCCCTTACTGGGCCTGGATTCCACCGGCTCCGGCGGCGGCAAGCCCATCCCCAACCCCTTACTGGGCCTGGATTCCACCGGCTCCGGCGGCGGCAAGCCCATCCCCAACCCCTTACTGGGCCTGGATTCCACCGGCTCCGGCGGCGGCAAGCCCATCCCCAACCCCTTACTGGGCCTGGATTCCACCGGCTCCGGCGGCGTCGACGGATCCgcggccgcaTAATGGGTTCGCGATAGGTCACTGATATATATCATAGCTTTTAGTTGTAGTCGGAAATCGTTTGACGTTTATTGTTTGTTCTCGGTGTGCCGTTAAAGTTTTCCCCCCTTTTTCTCTCTATTTGTTGAGTTCCATCGATGGACGGATCCGTGGACCTTTCAGAAGTATATAAATATATAACTAAAGAGTAACGAATTTGTCAGCGAATAAGGGGATTCCACATGCCACACTAACACAATGAAACTATGTACCTAACAGAGAAACTACATATGCATACACAGCAATACCGATAACCACTACCAATACCATTAGCCCACATAGACCCAAACTGGCCTAATCGATCTATAAAGCATATATAGTATATACTATTAGGGTCTCGCTTACGCGAATTTCTATTTACAAGCTCCGAAATCGAAACTGCAACACAAGATAGGGAAAGAACTGGAAATATATACTCAACACTTGTGTATATCATAAATCACATAATCCACACAAAGCCTATGATAATTATATTTATATCGGTATAATCGTATATACATATATAGCCAAAGATTGTTGTTGAACTGCGAACTGCAAGTTTTATTACCGTTGATGATATTATCTTCTGTGTAAAGAGAACATTTTAATCAAACCCAAGCAATTGTTGATGTTCGGCACGAATGATAACCATAAAAAGAACTAGCTAATCAAAAGTCATATGAATACTCTGTTAACTAATTACTGTTTATGTTTGTTGATATTATTTAATTCATTGATATTAATGCACCGTATGCTTTTGTGTTTAACTAACATGAGCAAGGCATGATGTAGGTACTAAAAATAATACTTAAGTAGATAGCCGTAGAAGCTTggctcgagcatggtcatagctgtttcctgtgtgaaattgttatccgctcacaattccacacaacatacgagccggaagcataaagtgtaaagcctggggtgcctaatgagtgagctaactcacattaattgcgttgcgctcactgcccgctttccagtcgggaaacctgtcgtgccagctgcattaatgaatcggccaacgcgcggggagaggcggtttgcgtattgggcgctcttccgcttcctcgctcactgactcgctgcgctcggtcgttcggctgcggcgagcggtatcagctcactcaaaggcggtaatacggttatccacagaatcaggggataacgcaggaaagaacatgtgagcaaaaggccagcaaaaggccaggaaccgtaaaaaggccgcgttgctggcgtttttccataggctccgcccccctgacgagcatcacaaaaatcgacgctcaagtcagaggtggcgaaacccgacaggactataaagataccaggcgtttccccctggaagctccctcgtgcgctctcctgttccgaccctgccgcttaccggatacctgtccgcctttctcccttcgggaagcgtggcgctttctcatagctcacgctgtaggtatctcagttcggtgtaggtcgttcgctccaagctgggctgtgtgcacgaaccccccgttcagcccgaccgctgcgccttatccggtaactatcgtcttgagtccaacccggtaagacacgacttatcgccactggcagcagccactggtaacaggattagcagagcgaggtatgtaggcggtgctacagagttcttgaagtggtggcctaactacggctacactagaagaacagtatttggtatctgcgctctgctgaagccagttaccttcggaaaaagagttggtagctcttgatccggcaaacaaaccaccgctggtagcggtggtttttttgtttgcaagcagcagattacgcgcagaaaaaaaggatctcaagaagatcctttgatcttttctacggggtctgacgctcagtggaacgaaaactcacgttaagggattttggtcatgagattatcaaaaaggatcttcacctagatccttttaaattaaaaatgaagttttaaatcaatctaaagtatatatgagtaaacttggtctgacagttagaaaaactcatcgagcatcaaatgaaactgcaatttattcatatcaggattatcaataccatatttttgaaaaagccgtttctgtaatgaaggagaaaactcaccgaggcagttccataggatggcaagatcctggtatcggtctgcgattccgactcgtccaacatcaatacaacctattaatttcccctcgtcaaaaataaggttatcaagtgagaaatcaccatgagtgacgactgaatccggtgagaatggcaaaagtttatgcatttctttccagacttgttcaacaggccagccattacgctcgtcatcaaaatcactcgcatcaaccaaaccgttattcattcgtgattgcgcctgagcgagacgaaatacgcgatcgctgttaaaaggacaattacaaacaggaatcgaatgcaaccggcgcaggaacactgccagcgcatcaacaatattttcacctgaatcaggatattcttctaatacctggaatgctgttttcccagggatcgcagtggtgagtaaccatgcatcatcaggagtacggataaaatgcttgatggtcggaagaggcataaattccgtcagccagtttagtctgaccatctcatctgtaacatcattggcaacgctacctttgccatgtttcagaaacaactctggcgcatcgggcttcccatacaatcgatagattgtcgcacctgattgcccgacattatcgcgagcccatttatacccatataaatcagcatccatgttggaatttaatcgcggcctagagcaagacgtttcccgttgaatatggctcatactcttcctttttcaatattattgaagcatttatcagggttattgtctcatgagcggatacatatttgaatgtatttagaaaaataaacaaataggggttccgcgcacatttccccgaaaagtgccacctgacgtctaagaaaccattattatcatgacattaacctataaaaataggcgtatcacgaggccctttcgtc
