# Supplementary material for: Multimerized epitope tags for high-sensitivity protein detection
Source: G3 (Bethesda). 2025 Apr 7;15(6):jkaf070. doi: 10.1093/g3journal/jkaf070 (PMC12134993; doi:10.1093/g3journal/jkaf070)
Supplement: jkaf070_Supplementary_Data [file jkaf070_supplementary_data.zip › Supplemental_Figure_Legends_and_Genotypes_G3-2025-405811.docx]

**Supplemental figure legends**

**Figure S1.** Assessment of conditionality of expression of vGlut-40XV5 and vGlut-40XMYC in adult brain. A-C) *vGlut-40XV5* germline excision. A) Synapsin; B) vGlut-40XV5; C) overlay. D-F) *B2RT-STOP-B2RT-vGlut-40XV5*. D) Synapsin; E) vGlut-40XV5; F) overlay. G-I) *vGlut-40XMYC* germline excision. G) Synapsin; H) vGlut-40XMYC; I) overlay. J-L) *B2RT-STOP-B2RT-vGlut-40XMYC*. J) Synapsin; K) vGlut-40XMYC; L) overlay. No expression is observed for either vGlut-40XV5 or vGlut-40XMYC in adult brains prior to excision of the STOP cassette. Brains in images A-F were immunostained, acquired, and processed identically. Brains in images G-L were immunostained, acquired, and processed identically. Scale bar: 100μm.

**Figure S2.**  Assessment of conditionality of expression of vGlut-40XV5 and vGlut-40XMYC at the third instar larval muscle 6/7 neuromuscular junction boundary. A-C) *vGlut-40XV5* germline excision. A) Synapsin; B) vGlut-40XV5; C) overlay. D-F) *B2RT-STOP-B2RT-vGlut-40XV5*. D) Synapsin; E) vGlut-40XV5; F) overlay. G-I) *vGlut-40XMYC* germline excision. G) Synapsin; H) vGlut-40XMYC; I) overlay. J-L) *B2RT-STOP-B2RT-vGlut-40XMYC*. J) Synapsin; K) vGlut-40XMYC; L) overlay. No expression is observed for either vGlut-40XV5 or vGlut-40XMYC at the larval neuromuscular junction prior to excision of the STOP cassette. NMJs in images A-F were immunostained, acquired, and processed identically. NMJs in images G-L were immunostained, acquired, and processed identically. Scale bar: 50μm.

**Figure S3.** Assessment of neuronal morphology in vGlut-40XV5 and vGlut-40XMYC larva with germline excisions of the STOP cassette. A-C) Brp immunostaining at larval NMJ muscle 6/7 boundary. A) *yw*; B) *vGlut-40XV5 GE*; C) *vGlut-40XMYC GE*. D-F) Dlg immunostaining at larval NMJ muscle 6/7 boundary. D) *yw*; E) *vGlut-40XV5 GE*; F) *vGlut-40XMYC GE*. G-I) Tdc2 immunostaining of larval ventral nerve cord. G) *yw*; H) *vGlut-40XV5 GE*; I) *vGlut-40XMYC GE*. J-L) FasII immunostaining of larval ventral nerve cord. J) *yw*; K) *vGlut-40XV5 GE*; L) *vGlut-40XMYC GE*. No obvious phenotypes are observed in the vGlut-40XV5 and vGlut-40XMYC germline STOP cassette excision genotypes as compared to *yw* controls. Scale bars: F-50μm, L-100μm.

**Supplemental figure genotypes**

**Figure S1.**A-C) *yw*; *B2RT-vGlut-40XV5 GE*/*B2RT-vGlut-40XV5 GE*; D-F) *yw*; *B2RT-STOP-B2RT-vGlut-40XV5*/*CyO,_Dfd-YFP_*; G-I) *yw*; *B2RT-vGlut-40XMYC GE*/*B2RT-vGlut-40XMYC GE*; J-L) *yw*; *B2RT-STOP-B2RT-vGlut-40XMYC*/*CyO,_Dfd-YFP_*.

**Figure S2.**A-C) *yw*; *B2RT-vGlut-40XV5 GE*/*B2RT-vGlut-40XV5 GE*; D-F) *yw*; *B2RT-STOP-B2RT-vGlut-40XV5*/*CyO,_Dfd-YFP_*; G-I) *yw*; *B2RT-vGlut-40XMYC GE*/*B2RT-vGlut-40XMYC GE*; J-L) *yw*; *B2RT-STOP-B2RT-vGlut-40XMYC*/*CyO,_Dfd-YFP_*.

**Figure S3.**A, D, G, J) *yw*; B, E, H, K) *yw*; *B2RT-vGlut-40XV5 GE*/ *B2RT-vGlut-40XV5 GE*; C, F, I, L) *yw*; *B2RT-vGlut-40XMYC GE*/ *yw*; *B2RT-vGlut-40XMYC GE*
